# Chronic Myelogenous Leukemia Cells Contribute to the Stromal Myofibroblasts in Leukemic NOD/SCID Mouse *In Vivo*

**DOI:** 10.1155/2012/901783

**Published:** 2012-07-15

**Authors:** Ryosuke Shirasaki, Haruko Tashiro, Yoko Oka, Takuji Matsuo, Tadashi Yamamoto, Toshihiko Sugao, Nobu Akiyama, Kazuo Kawasugi, Naoki Shirafuji

**Affiliations:** Department of Hematology/Oncology, Teikyo University School of Medicine, 2-11-1 Kaga, Itabashi-ku, Tokyo 173-8606, Japan

## Abstract

We recently reported that chronic myelogenous leukemia (CML) cells converted into myofibroblasts to create a microenvironment for proliferation of CML cells *in vitro*. To analyze a biological contribution of CML-derived myofibroblasts *in vivo*, we observed the characters of leukemic nonobese diabetes/severe combined immunodeficiency (NOD/SCID) mouse. Bone marrow nonadherent mononuclear cells as well as human CD45-positive cells obtained from CML patients were injected to the irradiated NOD/SCID mice. When the chimeric *BCR*-*ABL* transcript was demonstrated in blood, human CML cells were detected in NOD/SCID murine bone marrow. And CML-derived myofibroblasts composed with the bone marrow-stroma, which produced significant amounts of human vascular endothelial growth factor A. When the parental CML cells were cultured with myofibroblasts separated from CML cell-engrafted NOD/SCID murine bone marrow, CML cells proliferated significantly. These observations indicate that CML cells make an adequate microenvironment for their own proliferation *in vivo*.

## 1. Introduction

A bone marrow stromal tissue forms a specific environment, called niche, to generate and grow hematopoietic stem and progenitor cells [1, 2]. Bone marrow stroma is mainly created by a mesenchymal stem cell (MSC); however, a hematopoietic stem cell (HSC) and MSC have a crosstalk with each other. MSC is reported to differentiate into various kinds of cells such as chondrocytes, osteocytes, monocytes, adipocytes, and pericytes and which expresses CD29, CD44, CD71, CD90, CD105, CD106, CD166, Stro-1, and ICAM-1, but do not show CD34 and CD45 [3–5]. A fibroblast/myofibroblast (Fib), an important component that constructs a stroma, has been reported to be derived from MSC and not from HSC [6–10]. Recently, Shirai and colleagues reported that Fibs are also generated from HSCs in chronic myelogenous leukemia (CML) [11], given this finding and the traditional dogma that Fibs are of MSC origin, the specific origin of Fibs is controversial, and it is possible that normal Fibs originate from HSC [12].

Fibs are also reported to construct tumor niche. An endothelial cell surrounding a tumor expresses similar cell surface markers to that of an original tumor [13]. Experimentally, xenograft transplantation model with using an immunodeficiency mouse *in vivo* indicates that a chromosomal aberration observed in an original tumor is demonstrated in vascular endothelial cells when transplanted human melanoma cells and liposarcoma cells [14]. When lymphoma cells are transplanted to a mouse, intratumor vasculature shows similar chromosomal aberration to that of the original lymphoma cells [15]. MSCs are also reported to have a similar genetical abnormality to the original tumor [16]. Thus, it is possible that a tumor-forming cell can differentiate into a stromal cell.

Recently, we reported that bone marrow nonadherent mononuclear cells, collected from acute myelogenous leukemia (AML) patients with MLL-ELL translocation and CML patients, converted morphologically and functionally into myofibroblasts when observed primary long-term culture *in vitro* [17, 18]. We also observed that leukemic cells created a microenvironment for proliferation by converting to myofibroblasts.

Hematologic malignant disorders as well as normal hematopoiesis have been analyzed using nonobese diabetes/severe combined immunodeficiency (NOD/SCID) mouse system *in vivo* [19–21]. To determine whether CML cells differentiated into stromal myofibroblasts *in vivo*, CML cells were transplanted to NOD/SCID mice, and the stromal contribution of CML cells was observed.

## 2. Materials and Methods

### 2.1. Preparation of Cells

Our institutional ethical committee approved this study using clinical samples and experimental animals. Bone marrow cells were collected from informed CML patients admitted to our hospital and newly diagnosed with CML or from healthy volunteers. Samples were centrifuged in Ficoll-Paque (S.G. 1077, Lymphoprep, Fresenius Kabi Norge AS, Norway) to prepare a mononuclear cell fraction. One-day culture in Dulbecco's Modified Eagle's Medium (DMEM; Nissui, Japan) containing 10% fetal calf serum (FCS, CELLect GOLD; MP Biomedicals, Germany) eliminated an adherent cell-fraction, and a non-adherent mononuclear cell fraction was used for transplantation. Cells were also incubated with anti-human CD45 antibody-(Ab-) coated magnetic beads (Miltenyi Biotech, Germany) based on the manufacturer's instructions, and the separated cells were also used for transplantation [18].

### 2.2. *In Vivo* Transplantation to NOD/SCID Mice

NOD/SCID mice were purchased from Japan Charles River Inc., and mice were maintained in a specific pathogen-free room and were fed germ-free water and food containing antibiotics. After whole body irradiation of 2.5 Gray, mice were injected with non-adherent mononuclear cells from CML patients by tail vein. For the inactivation of NK cells, mice were injected intraperitoneally with antiasialo GM1 Ab (100 *μ*L/mice) (WAKO, Japan) prior to the transplantation and on each 11th day thereafter. Blood from mice were monitored for *BCR*-*ABL* fusion molecules with reverse transcription-polymerase chain reaction (RT-PCR). When the fusion mRNA was detected, mice were sacrificed and bone marrow cells and spleen cells were obtained. RNA was extracted from the indicated cells (Qiagen, CA, USA) the first strand cDNA was synthesized with an oligo-dT primer using a first-strand cDNA Synthesis Kit (Invitrogen, CA, USA), and RT-PCR was employed [22]. The primers used included major BCR 1st and 2nd, ABL 1st and 2nd, human CD13, CD33, CD34, CD133, CD106, fibroblast specific protein-1 (FSP1), vascular endothelial growth factor (VEGF) A, VEGF receptor type 1, type 2, and GAPDH [17, 18, 23, 24]. The PCR products were analyzed on a 2.5% agarose gel electrophoresis and were recovered with a GeneClean Kit (MD Biomedicals, OH, USA). A cDNA sequence of the fusion product was determined using a BigDye terminator v3.1 Cycle Sequencing Kit (Applied Biosystems (AB), CA, USA), and was analyzed with an ABI PRISM 3700 DNA analyzer (AB) [11].

### 2.3. Biological Characterization of the Engrafted CML Cells

Magnetic selections of the engrafted CML-derived fraction and CML-derived Fib-rich one were prepared with anti-human CD34 Ab-coated magnetic beads, and anti-human D7-FIB Ab-coated magnetic beads [25, 26], respectively, as per the manufacturer's direction (Miltenyi). The separated positive cells were cultured for one week, and the morphology was observed. To detect the transplanted human CML cells, anti-human CD13 Ab (Becton Dickinson (BD), CA, USA) and CD33 Ab (BD) were used. Anti-human CD133 Ab (Miltenyi), CD106 Ab (BD), were employed for the detection of human Fibs and analyzed with Cell Sorter (Beckman Coulter (BC), CA, USA). The procedures for immunocytochemical staining were reported previously [17, 18]. The antibodies used included anti-human smooth muscle actin (SMA) Ab (diluted with phosphate-buffered saline at 1 : 200, DAKO, Denmark), fibronectin Ab (1 : 200, Immunotech, BC), and FSP1 (also called S100) Ab (1 : 200, BD).

Fluorescent *in situ* hybridization (FISH) analysis was performed as reported previously, in which the 5′ portion of *BCR* and the 3′ part of *ABL* were labeled with SpectrumGreen (green) and SpectrumOrange (red), respectively (Abbott, IL, USA). Normal cells appeared split red and green signals, while cells having the translocation gave yellow because of the fusion of the 5′ and 3′ signals [18].

Cytokine production was assayed.The human VEGF-A immunoassay kit (Quantikine R&D Systems, MN, USA) was obtained commercially, and culturing supernatants after a 72-hour culture of the indicated myofibroblasts at 1 × 10^5^/mL in a 6-well plate (NUNC, NY, USA) that were priory starved for 24 hours were quantified according to the manufacturers' instructions [24].

Cell-proliferation was assayed. Non-adherent mononuclear cells were suspended in DMEM with 10% FCS to a concentration of 1 × 10^6^/mL for bone marrow cells from CML patients in the chronic (CP) and accelerated phase (AP), and 1 × 10^5^/mL for those in normal bone-marrow-derived CD34-positive cells and the blast crisis phase (BC). One hundred microliters of the cell suspension were cultured in a 96-well flat-bottomed plate (Corning Incorporated, Costar, NY, USA) with or without NOD/SCID murine bone-marrow-derived Fib layers, and anti-human VEGF-A-neutralizing Ab (Sigma, MO, USA). When cells were cultured on the feeder layers, indicated adherent Fibs (1 × 10^3^/well) were irradiated before-culture at 30 Gray (Hitachi MBR-15, Japan) [18], and the cell proliferation was assayed with a Cell Counting Kit (Dojindo, Japan) according to the manufacturer's instructions.

Parental CML cells or normal bone-marrow-derived non-adherent CD34-positive cells were also cultured in myofibroblast-layered 35 mm petri dishes (Corning Incorporated, Corning, NY, USA) for one day to adhere to the feeder layers, and dishes were gently washed three times to discard non-adherent cells. Cells were cultured for seven days, and the appearance was photographed. And cell foci (immature cell-clusters or colonies that adhered to the feeder layers after washing thoroughly) were counted microscopically.

### 2.4. Statistical Analysis

The data represent the mean ± standard error of the mean. The significance of differences among the groups was determined using Student's *t*-test, and is indicated with an asterisk (*P* < 0.01).

## 3. Results

### 3.1. Engraftment of the Injected Human CML Cells

**Figure 1:**
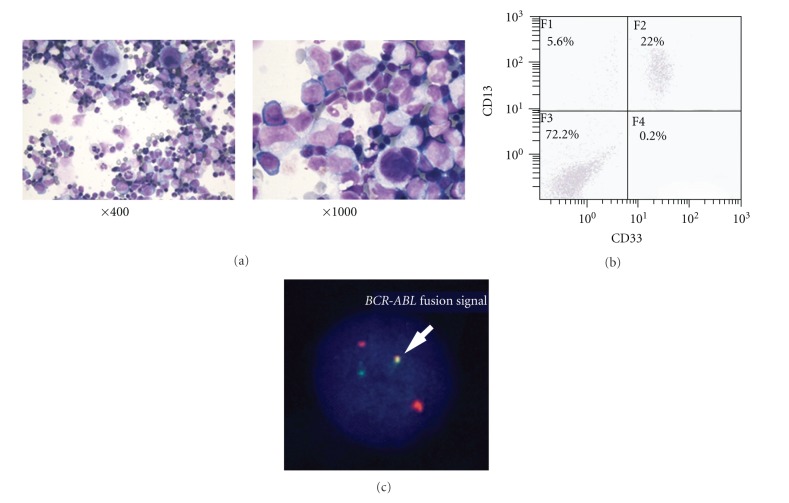
Characters of the transplanted CML-derived non-adherent mononuclear cells. (a) Smear slides of bone marrow cells obtained from CML patient (AP). Cells were stained with May-Giemsa solution (×400, and ×1000). (b) CD13 and CD33 expressions of the prepared non-adherent mononuclear cells from a CML (AP) patient. (c) FISH analysis. Normal cells appear split red (*ABL*) and green (*BCR*) signals, while cells having the translocation give yellow because of the fusion of the 5′ and 3′ signals. 95 cells out of 100 analyzed are positive.

Five CML patients (2 CP, 2 AP, and one myeloid BC) gave informed consent for the collection of bone marrow cells for analysis. Mononuclear cells were separated and cultured for one day to eliminate the adherent cell fraction, and the non-adherent fraction was removed and injected to NOD/SCID mice. Cells were also incubated with anti-human CD45 Ab-coated magnetic beads to eliminate stromal cells. Above 95% of non-adherent cells showed CD45 weakly positive or positive in all five cases. The morphology of the non-adherent cells from a patient of AP is shown in Figure 1(a). These cells expressed CD13 and CD33 (Figure 1(b), AP case). CD13/CD33 double positive cells were 12% and 18% in CP; 22% and 26% in AP; 81% in BC. In FISH analysis *BCR-ABL* fusion signal was observed (Figure 1(c)). *BCR-ABL* fusion signal-positive cells were 95 to 100% in all cases.

**Figure 2:**
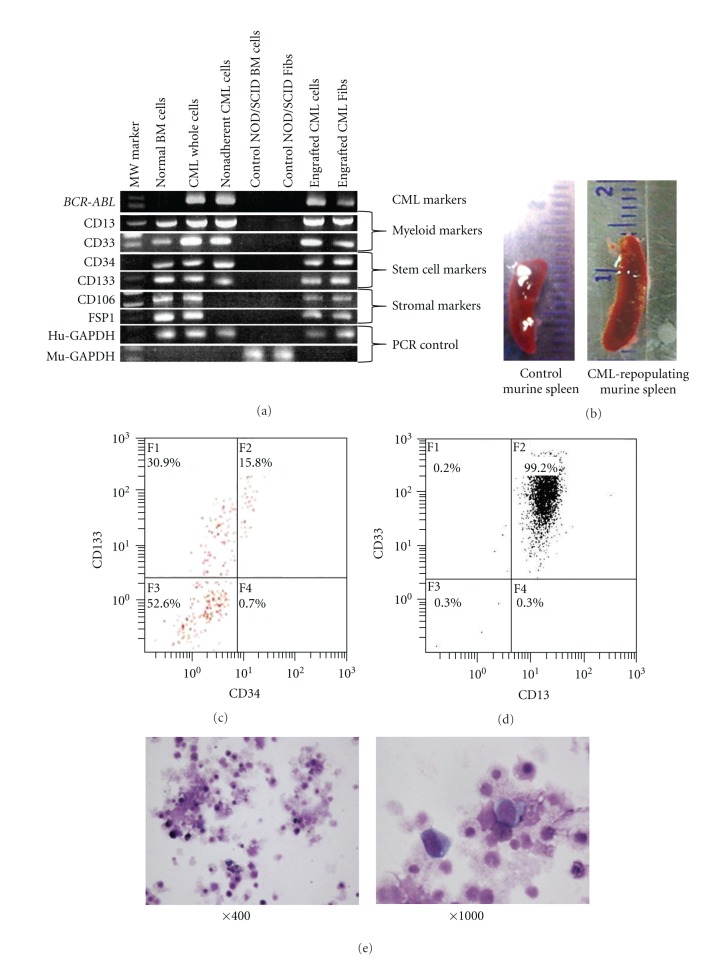
Analysis of the engrafted CML cells in the transplanted NOD/SCID murine bone marrow. (a) RT-PCR analyses of the indicated cells. Bcr-Abl was analyzed for the detection of fusion molecule; CD13 and CD33, human myeloid markers that are also expressed in the injected CML cells; CD34 and CD133, human stem cell markers that are expressed in the injected CML cells; CD106 and FSP1, human stromal markers that are expressed in human bone marrow adherent cells; GAPDH, RT-PCR control. MW indicates molecular weight marker; BM, bone marrow; CML whole, bone marrow mononuclear cells from CML patient with no processing; Fib, myofibroblasts. (b) Appearance of the spleen from the control NOD/SCID mouse and from human CML cell-engrafted NOD/SCID mouse. (c) CD34 and CD133 expressions of the CML cell-engrafted NOD/SCID murine bone marrow. (d) Expression of CD13 and CD33 after the engrafted NOD/SCID murine bone marrow cells were separated with anti-human CD34 Ab-coated magnetic beads. (e) Morphology of NOD/SCID murine bone marrow cells after engraftment of human CML cells. Cells were stained with May-Giemsa solution (×400, ×1000).

**Figure 3:**
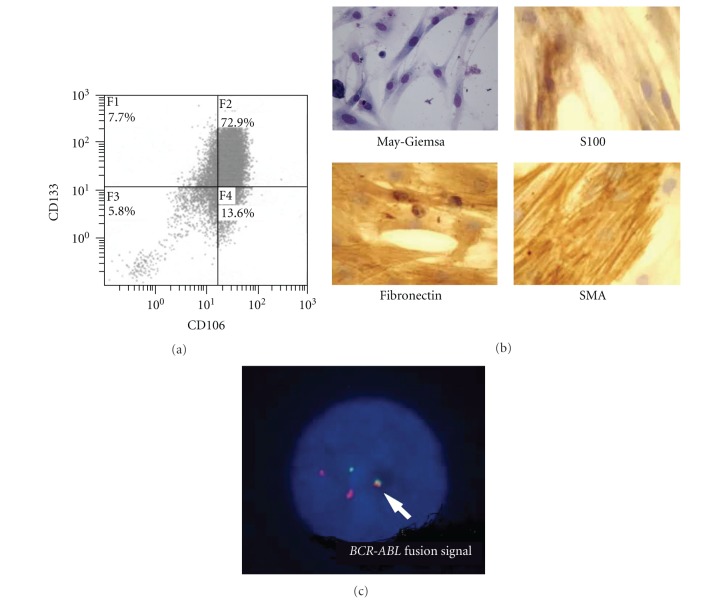
Characteristics of myofibroblasts from CML cell-engrafted NOD/SCID murine bone marrow cells. (a) Expression of CD106 and CD133. After selected with anti-human CD34 Ab-coated magnetic beads cells were further separated with anti-human D7-FIB Ab-coated magnetic beads, and analyzed. (b) Microscopical appearance of the separated myofibroblasts from CML cell-engrafted NOD/SCID murine bone marrow cells (×400). May-Giemsa, and immunocytochemical staining of S100, human fibronectin, and human SMA. (c) FISH analysis of the separated myofibroblasts from CML cell-engrafted NOD/SCID murine bone marrow. *BCR-ABL* fusion signal is indicated with an arrow. 83 cells are positive in total 100 cells analyzed.

The transplanted NOD/SCID mice were monitored their blood employing RT-PCR to identify Bcr-Abl fusion product that was expressed in the injected CML cells. After 2 months, Bcr-Abl fusion RNA was detected in blood (Figure 2(a)), and mice were sacrificed to detect human CML cells in murine bone marrow. Spleen was enlarged in CML cell-engrafted NOD/SCID mouse (Figure 2(b)). RT-PCR analysis yielded similar results for molecular expressions between injected CML cells and the engrafted CML cells (Figure 2(a)), in which CD13 and CD33 (human myeloid markers that were also expressed in the injected CML cells) and CD34 and CD133 (human stem cell markers that were expressed in the injected CML cells) were detected in the engrafted murine bone marrow cells. Also, CD106 and FSP1 (human stromal markers that were expressed in human bone marrow adherent cells) were expressed in the engrafted murine bone marrow. CML-engrafted NOD/SCID murine bone marrow cells expressed CD34 and CD133 at protein level (Figure 2(c)). To isolate human CML cells, CML cell-engrafted NOD/SCID murine bone marrow cells were incubated with anti-human CD34 Ab-coated magnetic beads. The microscopical morphology after separation is shown in Figure 2(e), in which human CD13 and CD33 double positive immature myeloid CML cells were observed in the transplanted NOD/SCID murine bone marrow (Figure 2(d)). To observe a contribution of the engrafted human CML cell-derived myofibroblasts to a NOD/SCID murine bone marrow stroma, separated CD34-positive cells were further selected with anti-human D7-FIB Ab-coated magnetic beads. The positive fraction after bead separation was analyzed with anti-human CD133 Ab and CD106 Ab (Figure 3(a)), in which 73% of the selected cells was double-positive for these two Abs when CML AP cells were injected. This double-positive cell fraction was 56% when CML CP cells were injected and 80% in BC cells, whereas below 1% was detected in normal human bone marrow cells. When the selected cells were cultured, cells were morphologically myofibroblasts, and similar to that of culturing Fibs *in vitro* (Figure 3(b)). Immunocytochemical staining revealed that the generated cells expressed human fibronectin, SMA, and S100, which are expressed in normal bone marrow myofibroblasts (Figure 3(c)). With FISH analysis *BCR-ABL* fusion gene was detected in these Fibs (Figure 3(d)). When CML cells (one CP and one AP) were selected with anti-human CD45 Ab-coated magnetic beads and transplanted to NOD/SCID mouse, CML cells were engrafted equally, and Fibs were also contributed to NOD/SCID murine bone marrow, in which CD133(+)CD106(+) cells occupied 65% (CP) and 82% (AP), respectively, in the total CD34(+)D7-FIB(+) cells.

 When non-adherent mononuclear cells prepared from CML patients were transplanted to NOD/SCID mice, engraftment was observed in 3 cases out of five (one CP, one AP, and one BC). The remaining two were dead because of bacterial infection at day 7 and day 8, respectively. When CD45-selected CML cells (one CP and one AP) were transplanted, two cases were engrafted.

### 3.2. Characterization of the Isolated CML-Derived Fibs

**Figure 4:**
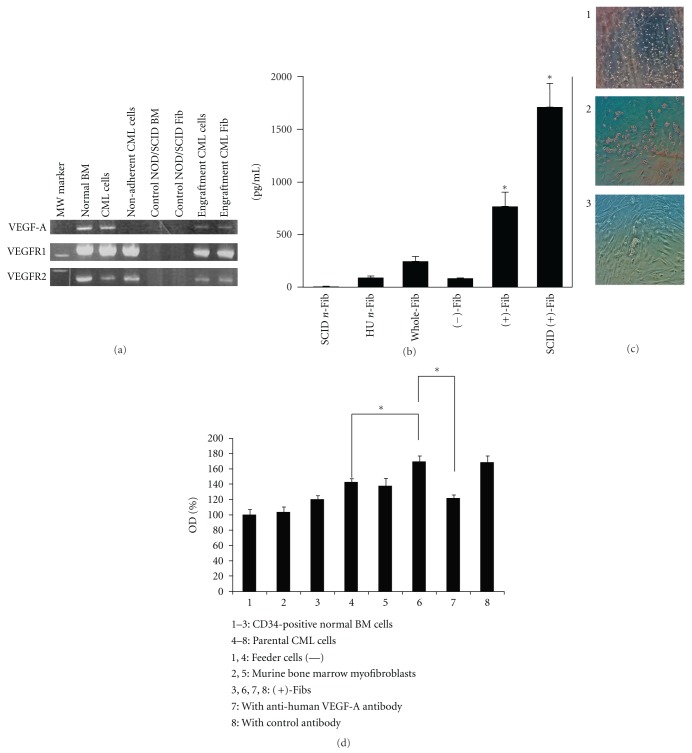
Functional analysis of the separated *BCR-ABL* positive myofibroblasts from CML cell-engrafted NOD/SCID murine bone marrow. (a) RT-PCR analysis of the indicated cells on VEGF-A system. (b) ELISA assay of human VEGF-A in the culturing supernatants. The values represent the mean % (*n* = 3) ± SD. SCID *n*-Fib, myofibroblasts from NOD/SCID murine bone marrow; Hu *n*-Fib, normal human bone-marrow-derived myofibroblasts; Whole-Fib, CML cell-derived myofibroblasts including *BCR-ABL* positive and negative cells that were prepared* in vitro*; (−)-Fib, CML bone marrow-derived *BCR-ABL* negative myofibroblasts prepared *in vitro*; (+)-Fib, CML bone marrow-derived *BCR-ABL* positive myofibroblasts prepared* in vitro*; SCID (+)-Fib, CML cell-engrafted NOD/SCID murine bone-marrow-derived *BCR-ABL* positive myofibroblasts. The asterisk of (+)-Fib indicates *P* < 0.01 between (+)-Fibs and other left side 4 Fibs, and the asterisk of SCID (+)-Fib indicates *P* < 0.01 between SCID (+)-Fibs and other left side 5 Fibs. (c) Cellular morphology when co-cultured with myofibroblasts. 1: Parental CML cells were cultured on the control NOD/SCID murine bone- bone marrow-derived myofibroblasts. 2: The parental CML AP cells were cultured on CML cell-engrafted NOD/SCID murine bone marrow-derived *BCR-ABL*-carrying SCID (+)-Fibs. 3: Normal bone marrow non-adherent CD34-positive cells were cultured on SCID (+)-Fibs (×200). (d) Cell-proliferation assay. 1–3: normal bone marrow-derived non-adherent CD34-positive cells, and 4–8: parental CML AP cells. 1, 4: No feeder layers, 2, 5: NOD/SCID murine bone marrow-derived stromal myofibroblasts, and 3, 6–8: CML cell-engrafted NOD/SCID murine bone marrow-derived *BCR-ABL* positive myofibroblasts. 7: Cultures are added with anti-human VEGF-A Ab, and 8: with control Ab. Asterisks indicate *P* < 0.01.

For the functional characterization, cytokine production was assayed using the supernatants of the cultured Fibs that carry *BCR-ABL* fusion chromosome (SCID (+)-Fibs). *In vitro* observation indicated that VEGF-A system plays important role in the proliferation of CML cells [18]. Thus, we focused on VEGF-A system.  Figure 4(b) shows the results, in which separated SCID (+)-Fibs derived from CML cell-engrafted NOD/SCID murine bone marrow produced human VEGF-A significantly compared with that of other Fibs (*P* < 0.01). Receptors for these cytokines were examined employing RT-PCR, in which the analyzed CML cells expressed VEGF receptor type 1, and type 2 (Figure 4(a)). To validate whether SCID (+)-Fibs stimulated to grow parental CML cells, CML cells as well as normal bone-marrow-derived CD34-positive cells were cultured on a feeder layer of Fibs, washed to completely discard cells that did not adhere to the Fibs, and further cultured (Figure 4(c)). When the parental CML cells were cultured on SCID (+)-Fibs, multiple leukemic cell foci were observed; however, when the parental CML cells were cultured on Fibs from control NOD/SCID mice, no blast foci were noted and matured cells were observed to be floating (Figure 4(c)-(1)). When normal bone-marrow-derived CD34-positive non-adherent mononuclear cells, immature hematopoietic cells at HSC level were cultured on SCID (+)-Fibs, a few cell foci were observed (Figure 4(c)-(3)). Cell foci were microscopically counted, in which 11 foci were counted in CD34-positive cell-input group, whereas 42 foci in CML cell group. No cell-foci were observed when CML cells were cultured with control NOD/SCID murine-bone marrow-derived Fibs. To validate VEGF-A-mediated activation in this system, cell-proliferation was assayed, in which the parental CML cells showed growth-promoting activity when cells were cultured with SCID (+)-Fibs. And when human VEGF-A neutralizing Ab was added to the cultures, cell-growth was inhibited (*P* < 0.01) (Figure 4(d)).

## 4. Discussion

We have shown in the present study that CML cells from the patients' bone marrow were engrafted in NOD/SCID mice to induce leukemia, in which human CML cells generated stromal myofibroblasts in NOD/SCID murine bone marrow, and functionally similar to that of CML cells generated *in vitro* in terms of their production of VEGF-A, and in supporting the proliferation to the parental CML cells.

To recapture the characteristics of *de novo* CML, the leukemic NOD/SCID mouse system was used. NOD/SCID mouse maintained the *de novo* CML characters without any cytokine supplementation in our experiment. We previously reported that VEGF-A system played an important role in the creation of microenvironment for proliferation of CML cells *in vitro* [18]. VEGF is one of important cytokines that regulate normal and abnormal hematopoiesis. Several reports indicate that VEGF-A system plays an important role in proliferation of hematological malignant cells with an autocrine or paracrine fashion [24, 27–30]. We identified that CML cell-derived stromal Fibs produced a high amount of VEGF-A *in vitro* and also *in vivo*. And CML cells expressed VEGF-A receptors. When the parental CML cells were cultured on SCID (+)-Fib *in vivo*, blastic CML cells grew significantly; however, when the parental CML cells were cultured on the control NOD/SCID murine bone-marrow-derived Fibs, expansion of immature cells were very low level, and cells were differentiated. Normal human bone-marrow-derived immature cells also grew on SCID (+)-Fib to make a few cell foci. These observations indicate that CML cells differentiate and create an adequate microenvironment for their growth and may behave like nonmutated HSCs. Further investigations will reveal the real characteristics of CML stem cells and their myofibroblastic conversion.

## 5. Conclusion

When CML cells were transplanted and engrafted to the irradiated NOD/SCID mice, CML cell-derived *BCR-ABL*-carrying myofibroblasts composed with NOD/SCID murine bone marrow stroma and made an adequate microenvironment for proliferation of parental CML cells *in vivo*.
